# Myotubularin-related protein protects against neuronal degeneration mediated by oxidative stress or infection

**DOI:** 10.1016/j.jbc.2022.101614

**Published:** 2022-01-29

**Authors:** Supender Kaur, Yu Sang, Alejandro Aballay

**Affiliations:** Department of Molecular Microbiology & Immunology, Oregon Health & Science University, Portland, Oregon, USA

**Keywords:** *Caenorhabditis elegans*, myotubularin, *Pseudomonas aeruginosa*, pathogens, infection, dendrite degeneration, AD, Alzheimer’s disease, NDs, neurodegenerative diseases, NGM, nematode growth medium, RFP, red fluorescent protein, RNAi, RNA interference

## Abstract

Microbial infections have been linked to the onset and severity of neurodegenerative diseases such as amyotrophic lateral sclerosis, multiple sclerosis, Alzheimer's disease, but the underlying mechanisms remain largely unknown. Here, we used a genetic screen for genes involved in protection from infection-associated neurodegeneration and identified the gene *mtm-10*. We then validated the role of the encoded myotubularin-related protein, MTM-10, in protecting the dendrites of *Caenorhabditis elegans* from degeneration mediated by oxidative stress or *Pseudomonas aeruginosa* infection. Further experiments indicated that *mtm-10* is expressed in the AWC neurons of *C. elegans*, where it functions in a cell-autonomous manner to protect the dendrite degeneration caused by pathogen infection. We also confirm that the changes observed in the dendrites of the animals were not because of premature death or overall sickness. Finally, our studies indicated that *mtm-10* functions in AWC neurons to preserve chemosensation after pathogen infection. These results reveal an essential role for myotubularin-related protein 10 in the protection of dendrite morphology and function against the deleterious effects of oxidative stress or infection.

The increase in life expectancy that occurred in the last century has been accompanied by an increase in neurodegenerative diseases (NDs). While specific factors are responsible for NDs emerge, it is generally accepted that their causes are mainly multifactorial. Underlying causes of NDs include aging, genetic mutations, environmental factors, misfolded proteins, and cellular dysfunctions like mitochondrial and oxidative stress. In addition, pathogenic microorganisms may also play a role in the onset or severity of NDs (1, 2). Microorganisms can induce neurodegeneration upon infection through different mechanisms. They can affect neurotransmitter levels and various cell signaling proteins that cause inflammation, pass the blood-brain barrier, and produce hallmarks of neurodegenerative disorders. Furthermore, the microorganisms present in the gut can have positive and negative effects on various NDs (3), and gut microbiota has been shown to regulate neuroinflammation in Parkinson’s disease (4). *Chlamydia* *pneumoniae* infection has been implicated in the advancement of Alzheimer’s disease (AD), meningoencephalitis, and multiple sclerosis (5), whereas infections by the Herpes Simplex Virus Type 1 and HIV have been implicated in AD (6, 7).

Neuronal cells extend into an axon and several dendrites that protrude from their cell bodies. Dendrites have distinct structural and functional properties that enable them to receive information from the environment and interact with the other neurons. Studies have shown the loss of dendritic complexity and withering in diseases like AD, stroke, and depression (8, 9, 10). There is a strong correlation between the severity of dendrite loss and behavioral deficits. To understand the role of pathogen infection in dendrite degeneration and behavioral deficiencies, we used the nematode *Caenorhabditis elegans*. *C. elegans* is an attractive model to study NDs because of its distinctive attributes, such as the well-characterized genetic toolsets and the mapping of the entire neural wiring of the organism (11). Moreover, studies in *C. elegans* have demonstrated the role of pathogens in inducing neural changes considered hallmarks of neurodegeneration (12, 13, 14, 15).

In this study, we identified a mutation in a myotubularin-related protein (MTMR), MTM-10, that results in enhanced susceptibility to pathogen-induced dendrite degeneration in *C. elegans*. Myotubularins are part of a disease-associated family of phosphatases that act on phosphoinositides (16, 17). They play critical roles in cell proliferation, regulation of actin structure, endocytosis, phagosome maturation, and cell survival. They have been linked with Charcot-Marie-Tooth neuropathies, which are characterized by defects primarily arising in myelin, axons, or both (16, 18, 19, 20). MTMR10, in particular, is present in various tissues and highly enriched in the brain (Human Protein Atlas proteinatlas.org) (21). We found that the *mtm-10* gene is expressed in olfactory AWC neurons of *C. elegans*, where it functions to protect the animals from dendrite degeneration caused by oxidative stress or pathogen infection. Our findings indicate a role of *mtm-10* in protecting animals from stress-induced dendrite degeneration in a cell-autonomous manner, providing a mechanistic link between *mtm-10* and dendrite degeneration.

## Results

### *mtm-10* is required to prevent pathogen-induced dendrite degeneration

**Figure 1:**
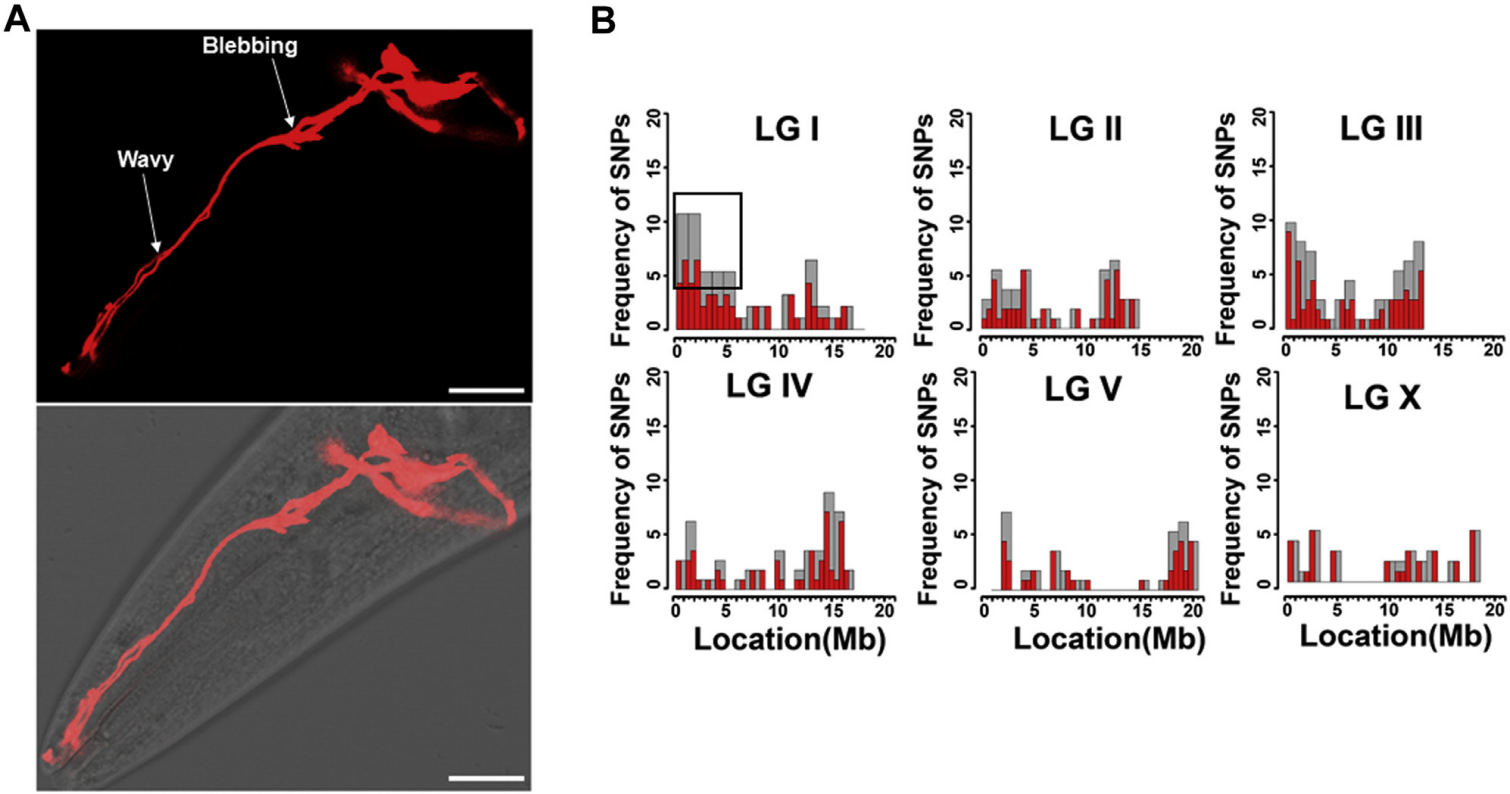
**Forward genetic screen to isolate mutants susceptible to pathogen-induced dendrite degeneration.***A*, representative photomicrograph of dendrite degeneration in AY161 mutant expressing RFP under the *odr-1* promoter after exposure to *Psuedomonas aeruginosa* for 24 h. The scale bars represent 10 μm. Different morphological changes in the dendrites are indicated with *white arrows*. *B*, linkage analysis of SNPs for mutant AY161 showed linkage around gene *Y48G1C.10* located in chromosome I of *Caenorhabditis elegans*. RFP, red fluorescent protein.

To identify genes that play a role in the pathogen-induced neurodegeneration, we conducted a forward genetic screen for mutants exhibiting enhanced susceptibility to *Pseudomonas aeruginosa* infection (13). The strain used for the screening was CX5974, which expresses red fluorescent protein (RFP) in AWC, AWB, and I1 neurons under the *odr-1* promoter (22). We selected 17 mutants from a screen of approximately 80,000 mutagenized haploid genomes showing significant changes in the morphology of the dendrites ((13) and Fig. S1). In *C. elegans*, the morphological changes in the dendrites of sensory or motor neurons are used as readouts for neuronal dysfunction or neurodegeneration (23). These changes in the dendrites include blebbing, waviness, and formation of bead-like structures along the length of the dendrites. Mutant AY161 showed significant changes in the morphology of the dendrites of the AWC neurons, including dendrite blebbing and wavy-like structures (Fig. 1*A*). These changes, hallmarks of dendrite degeneration, were observed only after 24 h of exposure to *P. aeruginosa* compared to control animals.

**Figure 2:**
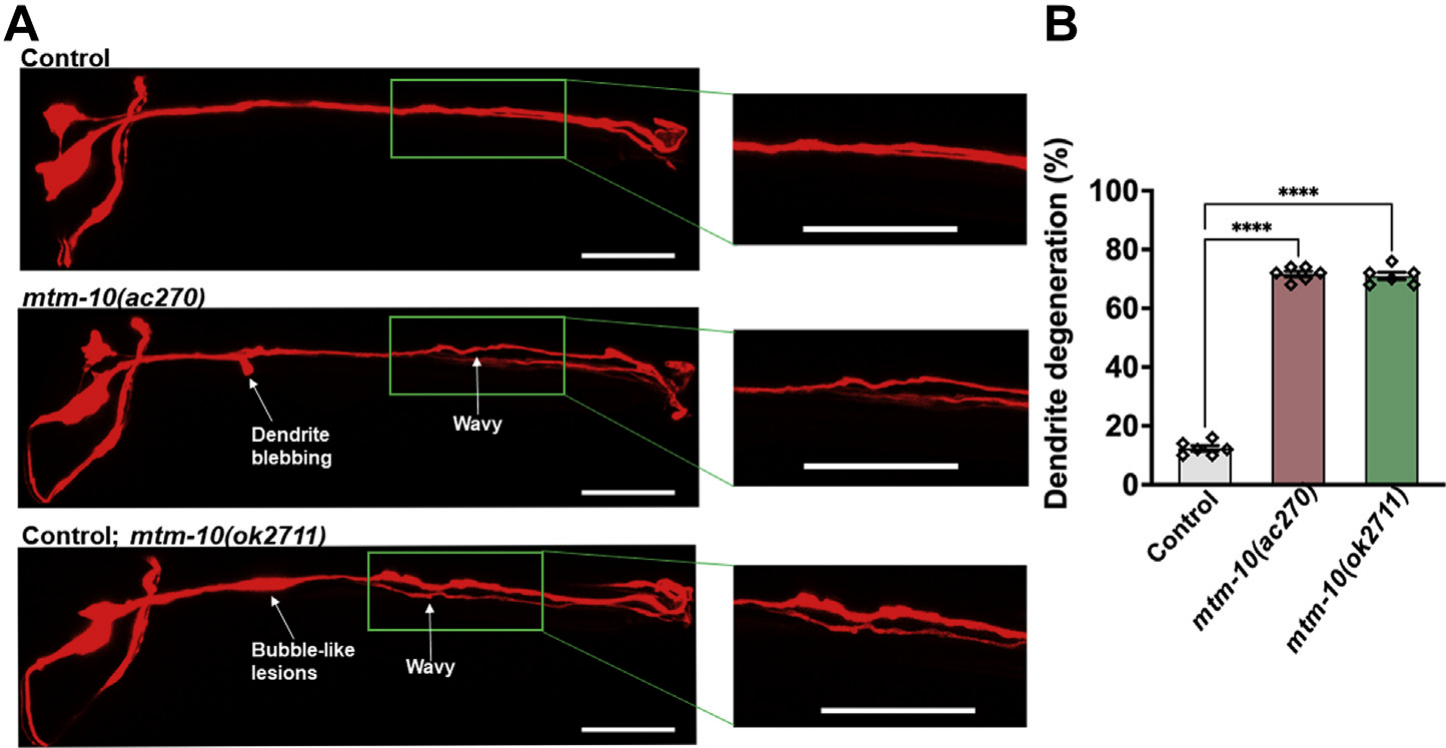
***mtm-10* animals are susceptible to pathogen-induced dendrite degeneration.***A*, representative photomicrographs of dendrite degeneration in control CX5974 (parent strain), *mtm-10 (ac270)*, and *mtm-10(ok2711)* animals expressing RFP in AWC after exposure to *Pseudomonas aeruginosa* for 24 h. The scale bars represent 10 μm and 2 μm, respectively. Different morphological changes in the dendrites are indicated with *white arrows*. *B*, quantification of dendrite degeneration in control CX5974 (parent strain), *mtm-10 (ac270)*, and *mtm-10(ok2711)* animals expressing RFP in AWC. Control *versus mtm-10(ac270)* and Control *versus mtm-10(ok2711)* (*p* < 0.0001) *via* one-way ANOVA test. The *black symbols* represent individual data points. (N = 6, number of animals = 50). ∗∗∗∗*p* < 0.0001. RFP, red fluorescent protein.

After subtracting common variants, linkage maps of SNPs were obtained (Fig. 1*B*). We focused on 4 Mb, defined as the mapping region for chromosome I, which shows mutations in genes *Y48G1C.10*, *F47G6.3*, and *unc-89*. Priority was given to *Y48G1C.10* because myotubularin-related proteins have been linked to peripheral neuropathy in mice (24). Further analysis of the mapped region of mutant AY161 revealed a single G→A mutation in the *Y48G1C.10* gene, resulting in a glycine to alanine substitution at residue 270. The G270A mutation is in a conserved domain also present in MTMR10 (Fig. S2). Also, the G270 site is conserved among several MTMR family proteins (Fig. S3). Based on the orthology aggregation prediction (Fig. S4) (DRSC Integrative Ortholog Prediction Tool; http://www.flyrnai.org/diopt) (25), gene *Y48G1C.10* was named *mtm-10*. To confirm that the aforementioned phenotypes of *mtm-10(ac270)* animals were because of mutation in the *Y48G1C.10* gene, we also studied *mtm-10(ok2711)* animals, which carry a 575-bp deletion that ranges from 2875 bp to 3449 bp in the genomic DNA. As shown in Figure 2, both *mtm-10(ok2711)* and *mtm-10(ac270)* animals exhibited dendrite degeneration after 24 h of exposure to *P. aeruginosa*. Overall, the percentage of the animals exhibiting the dendrite degeneration phenotype was 72% for *mtm-10*(*ac270*), 70% for *mtm-10(ok2711)*, and only 12% for control animals (Fig. 2*B*).

We also studied whether mutation in *mtm-10* makes the animals susceptible to dendrite degeneration to an insult other than pathogen infection. We exposed control and mutant animals to 5 mM paraquat for 24 h to cause oxidative stress. After a 24 h treatment, mutant *mtm-10(ac270)* and the deletion strain *mtm-10(ok2711)* exhibited morphological changes in the dendrites of the neurons (Fig. S5*A*). In addition, expression of *mtm-10* under the control of its own promoter rescued the paraquat-induced dendrite degeneration phenotype of *mtm-10(ac270)* animals (Fig. S5*B*). Taken together, these results suggest that *mtm-10* may protect the neurons of *C. elegans* from dendrite degeneration induced by stress in general.

### Cell-autonomous role of *mtm-10(ac270)* in pathogen-induced dendrite degeneration

**Figure 3:**
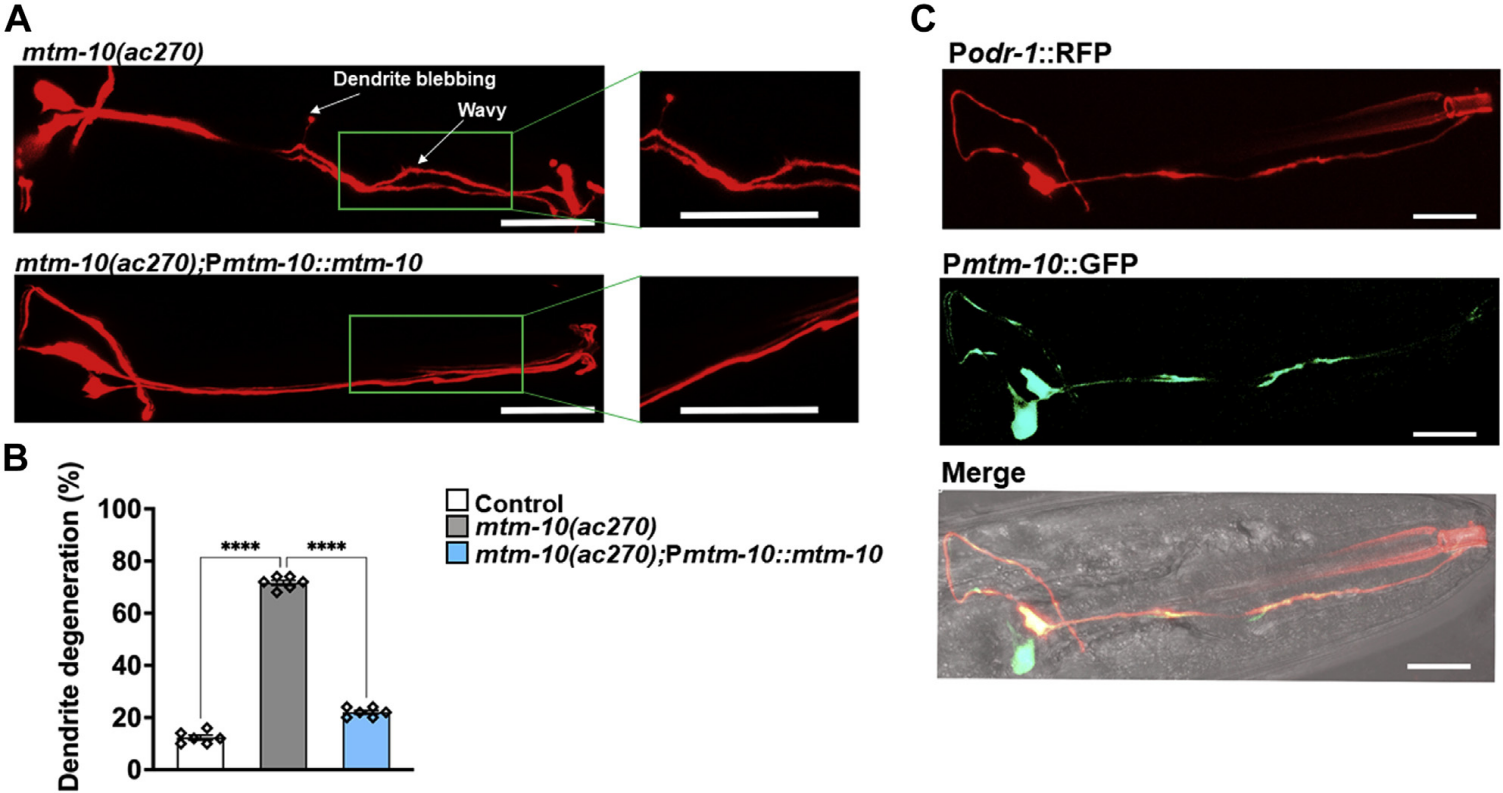
***mtm-10* gene expression rescues the pathogen-induced dendrite degeneration phenotype of *mtm-10* mutants.***A*, representative photomicrographs of dendrite degeneration after exposure to *Pseudomonas aeruginosa* for 24 h in *mtm-10(ac270)* animals and animals expressing *mtm-10* under its own promoter *mtm-10(ac270)*; P*mtm-10*::*mtm-10*. In both cases, animals also express RFP in AWC neurons. The scale bars represent 10 μm and 2 μm, respectively. Different morphological changes in the dendrites are indicated with *white arrows*. *B*, quantification of the dendrite degeneration phenotype in control CX5974 (parent strain), *mtm-10(ac270)*, and *mtm-10(ac270)*;P*mtm-10::mtm-10* animals expressing RFP in AWC neurons. The *black symbols* represent individual data points. N = 6, number of animals = 50. Control *versus mtm-10(ac270)* (*p* < 0.0001) and *mtm-10(ac270) versus mtm-10(ac270)*;P*mtm-10::mtm-10* (*p* < 0.0001) *via* one-way ANOVA test. *C*, representative photomicrographs of animals expressing RFP under the control of the *odr-1* promoter and GFP under the control of the *mtm-10* promoter. The scale bars represent 5 μm. ∗∗∗∗*p* < 0.0001. RFP, red fluorescent protein.

To further confirm that mutation in gene *mtm-10(ac270)* is the cause of the animal’s susceptibility to pathogen-induced dendrite degeneration, we created *mtm-10(ac270)* animals expressing *mtm-10* under its endogenous promoter. As shown in Figure 3, *A* and *B*, the phenotype of *mtm-10(ac270)* animals was completely rescued by the WT gene expression.

**Figure 4:**
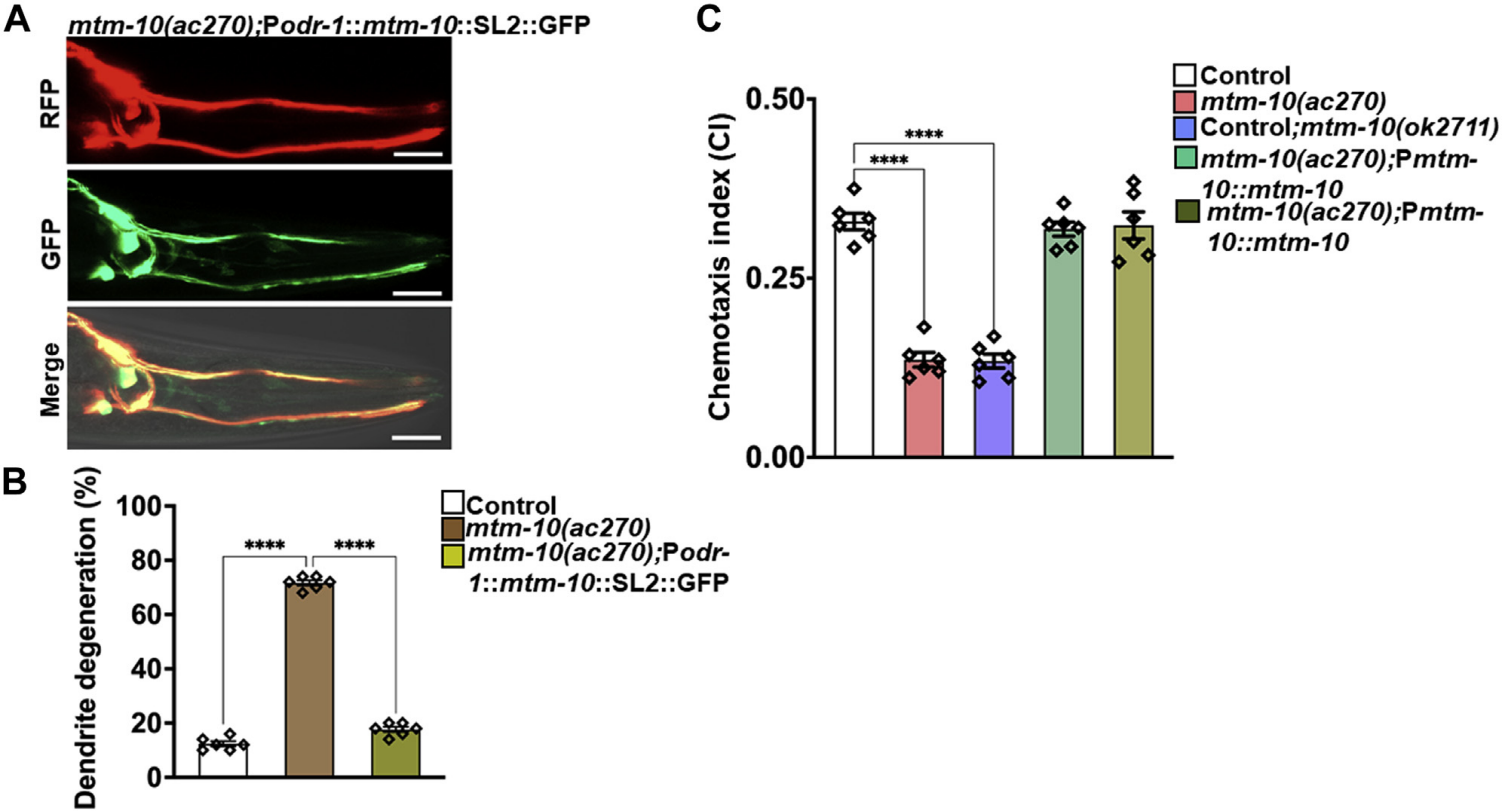
***mtm-10* mutation affects the function of AWC neurons in a cell-autonomous manner.***A*, representative photomicrographs of *mtm-10* animals expressing *mtm-10* and GFP under the control of the *odr-1* promoter after 24 h of *Pseudomonas aeruginosa* infection. The scale bars represent 5 μm. *B*, quantification of the dendrite degeneration phenotype in control CX5974 (parent strain), *mtm-10(ac270)*, and *mtm-10(ac270)*;P*odr-1*::*mtm-10*::SL2::GFP animals. The *black symbols* represent individual data points. (N = 6, number of animals = 50). Control *versus mtm-10(ac270)*(*p* < 0.0001) and *mtm-10(ac270) versus mtm-10(ac270)*;P*odr-1*::*mtm-10*::SL2::GFP(*p* < 0.0001) *via* one-way ANOVA test. *C*, quantification of chemotaxis index (CI) in control CX5974 (parent strain), *mtm-10(ac270)*, *mtm-10(ok2711)*, *mtm-10(ac270)*;P *mtm-10::mtm-10*, and *mtm-10(ac270)*;P*odr-1*::*mtm-10*::SL2::GFP animals in response to benzaldehyde. Control *versus mtm-10(ac270)*, (*p* < 0.01) and Control *versus mtm-10(ok2711)*, (*p* < 0.0001), *via* one-way ANOVA test. The *black symbols* represent individual data points (N = 6). ∗∗∗∗*p* < 0.0001. RFP, red fluorescent protein.

Subsequently, we decided to study the expression pattern of gene *mtm-10(ac270)* by expressing GFP driven by the 4-kb promoter of *mtm-10(ac270)* in WT animals. We also drove the expression of RFP to AWC neurons using the *odr-1* promoter. The transgene *mtm-10*::GFP was expressed in head neurons, and the expression colocalized with the RFP expressing AWC neurons (Fig. 3*C*). In addition, GFP was also observed in a head neuron other than AWC (Fig. 3*C*). Thus, we reasoned that *mtm-10(ac270)* could have cell-autonomous or cell nonautonomous effects in pathogen-induced dendrite degeneration. We used 4 kb of the *odr-1* promoter to express *mtm-10(ac270)* only in AWC neurons to distinguish between these possibilities, As shown in Figure 4, *A* and *B*, *mtm-10(ac270)* animals expressing *mtm-10(ac270)* under the *odr-1* promoter showed complete rescue of the pathogen-induced dendrite degeneration phenotype. These results suggest *mtm-10(ac270)* functions cell-autonomously and protects AWC neurons from the dendrite degeneration caused by *P. aeruginosa* infection.

### Impairment of chemosensation by pathogen-induced dendrite degeneration

Chemosensory neurons play an essential role in chemosensation used by *C. elegans* to avoid noxious conditions and locate food. Any defect in cilium structure or connection between the cilia and the cell body results in defective chemosensation (26). *C. elegans* are attracted to several volatile organic compounds as these are natural products of bacterial metabolism (27). Chemosensory AWC neurons are required for chemotaxis driven by odors like benzaldehyde, butanone, isoamyl alcohol, 2,3-pentanedione, and 2,4,5-trimethylthiazole.

To assess the effect of pathogen-induced dendrite degeneration on the functionality of AWC neurons, we performed a chemotaxis assay. This assay was performed 24 h post-infection using benzaldehyde as a chemical attractant sensed by both right and left AWC neurons (28). As shown in Figure 4*C*, both *mtm-10(ac270)* and *mtm-10(ok2711)* animals exhibited a significant reduction in the chemotaxis index compared to control animals. The expression of *mtm-10* under its own promoter or the *odr-1* promoter fully rescued the chemotaxis defect of *mtm-10(ac270)* animals (Fig. 4*C*). These results indicate that pathogen infection in both *mtm-10 (ac270)* and *mtm-10(ok2711)* affects neural morphology and impairs AWC function.

### Pathogen exposure reduces longevity in *mtm-10* animals and the increase in expression of DAF-16 regulated genes

**Figure 5:**
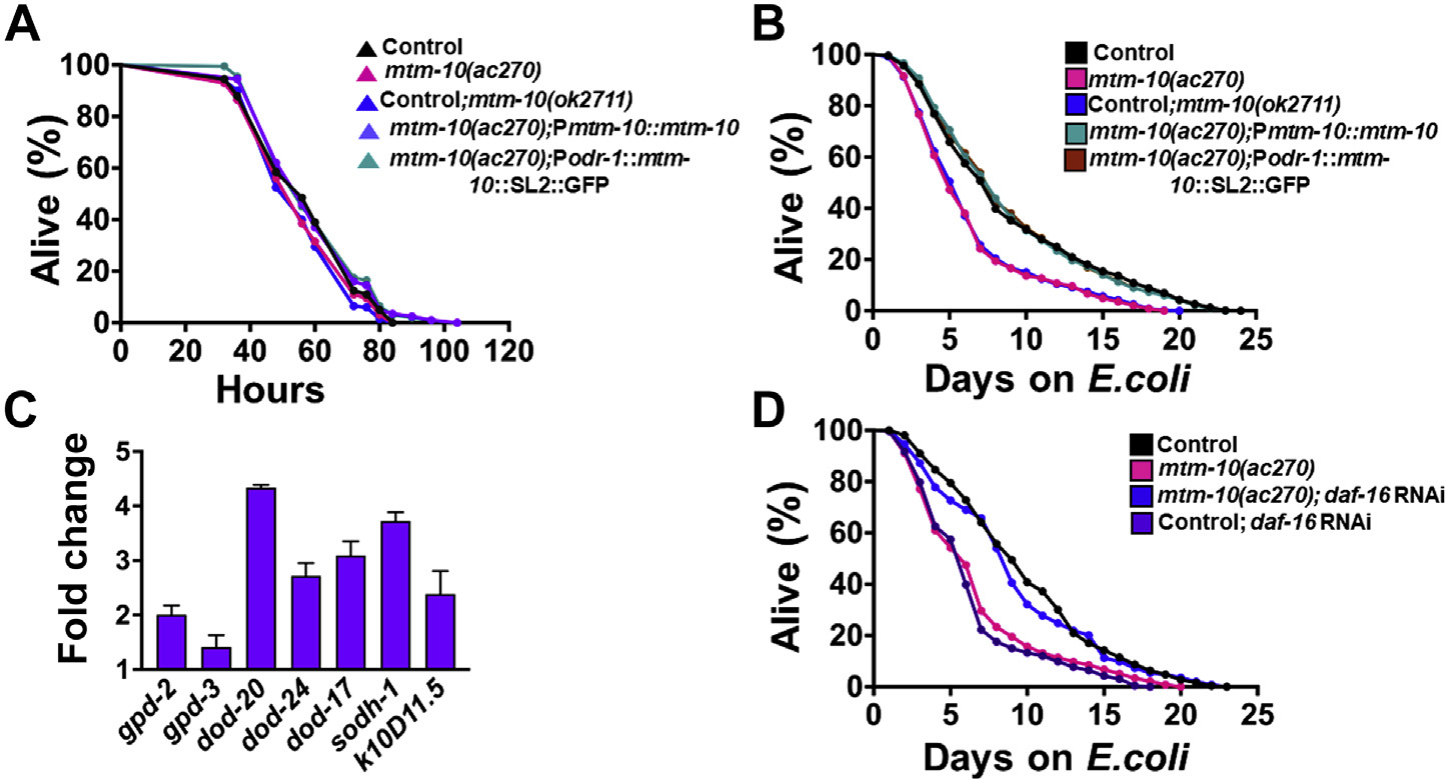
***mtm-10* mutation enhances susceptibility to pathogen-induced dendrite degeneration and decreases the lifespan.***A* representative survival plot of control CX5974 (parent strain), *mtm-10(ac270)*, *mtm-10(ok2711)*, *mtm-10(ac270)*;P*mtm-10*::*mtm-10*, and *mtm-10(ac270)*;P*odr-1*::*mtm-10*::SL2::GFP animals on full lawns of *Pseudomonas aeruginosa* (independent replicates N = 3, number of animals n = 200), control *versus mtm-10 (ac270)*(n.s.). *B*, representative lifespan plots of control CX5974 (parent strain), *mtm-10(ac270)*, *mtm-10(ok2711)*, and rescued strains *mtm-10(ac270)*; P*mtm-10::mtm-10*, and *mtm-10(ac270)*;P*odr-1*::*mtm-10*::SL2::GFP on UV-killed *Escherichia coli OP50* (independent replicates N = 3, number of animals n = 300), control *versus mtm-10(ac270)*, (*p* < 0.0001). *C*, quantitative real-time PCR analysis of DAF-16-dependent genes in *mtm-10(ac270)* animals compared with control CX5974 (parent strain) after infection with *P. aeruginosa* for 24 h (n = 3; three technical replicates in each case). *D*, representative lifespan plots of control CX5974 (parent strain) and *mtm-10(ac270)* animals after knockdown of *daf-16 via* RNAi followed by 24-h exposure to *P. aeruginosa* (n = 3; animals per condition = 300). *mtm-10(ac270)* control RNAi *versus mtm-10(ac270)*; *daf-16* RNA (*p* < 0.0001). RNAi, RNA interference.

We asked whether degeneration in AWC dendrites makes the animals more susceptible to *P. aeruginosa*. We observed no significant change in the survival of control and *mtm-10(ac270) animals* constantly exposed to *P. aeruginosa* (Fig. 5*A*), indicating that *mtm-10(ac270)* animals are not more susceptible to pathogen infection than control animals. These results also suggest that dendrite degeneration is not the result of the premature death of the animals due to increased susceptibility to pathogens.

We then asked whether a short infection by *P. aeruginosa* may alter the longevity of *mtm-10(ac270)* animals. We performed an acute *P. aeruginosa* infection by exposing the animals to the pathogen for only 24 h. The animals were rinsed with a buffer containing antibiotics to remove any live *P. aeruginosa* before transferring them to plates that contained antibiotics and were seeded with UV-killed *Escherichia coli* (29). As shown in Figure 5*B*, there was a significant decrease in the lifespan of *mtm-10(ac270)* animals infected for 24 h with *P. aeruginosa* compared to control animals. As shown in Figure 5*B*, expression of *mtm-10* fully rescued the decreased longevity of *mtm-10(ac270)* animals caused by pathogen infection, indicating that *mtm-10* plays a role in the control of pathways regulating longevity.

Because decreased longevity in animals susceptible to pathogen-induced degeneration is because of uncontrolled activation of immune genes regulated by the FOXO family transcription factor DAF-16 that is part of the insulin/insulin-like growth factor-1 receptor homolog DAF-2 (13), we studied the expression of DAF-16-regulated genes in *mtm-10(ac270)* animals. As shown in Figure 5*C*, the DAF-16 regulated genes were upregulated in *mtm-10(ac270)* animals compared to control animals, suggesting that the uncontrolled activation of DAF-16 may be responsible for the decreased lifespan of *mtm-10* animals exposed to *P. aeruginosa* during the young adult stage. Consistent with the idea that uncontrolled activation of DAF-16 is the reason for the decreased lifespan of *mtm-10(ac270)* animals, RNA inhibition of DAF-16 rescued the decreased lifespan of *mtm-10(ac270)* animals infected with *P. aeruginosa* early in life (Fig. 5*D*).

## Discussion

The societal burden of NDs increases annually, and there is a need for technologies that can accelerate research to identify new targets and develop novel therapeutics. Neurodegenerative diseases occur through the loss of neurons and neuronal function, the disappearance of neuron cell bodies, broken axons or dendrites, dendrite beading/blebbing, and an increase in the expression of pathogenic proteins (30, 31, 32). Dendrites have an essential role in integrating neuronal information as they are precisely patterned across the nervous system. Dendrite degeneration is associated with aging, injury, and the pathology of NDs (33, 34, 35, 36).

Myotubularin-related proteins are part of a prominent disease-associated family, and their loss of function has been reported in the degeneration of sensory and motor neurons (37, 38). This family encompasses catalytically active and inactive phosphatases (39), which are well conserved from humans to nematodes (40). Extensive clinical and genetic data have linked mutations in MTMRs with Charcot-Marie-Tooth disease (41) and myotubular myopathy (42), characterized by muscle weakness and impairment in the maturation of muscle fibers. While most neuropathies related to myotubularin mutations are caused by defects in axons, myelin, or both, loss of myotubularin in the Schwann cells is also sufficient to cause neuropathy in mammals (43).

We found that the animals with a mutation in the myotubularin–related gene *mtm-10* were more susceptible to oxidative stress and pathogen-induced dendrite degeneration. We also found that dendrite degeneration in AWC neurons also affected neuronal function and that pathogen-induced dendrite degeneration negatively impacted the lifespan of *mtm-10(ac270)* animals. We observed that genes controlled by DAF-16 were upregulated in *mtm-10(ac270)* animals and that the decrease in lifespan was rescued by knockdown of *daf-16* in the *mtm-10(ac270)* animals, suggesting that the excessive activation of DAF-16 resulted in a reduced lifespan in those animals.

In summary, our study suggests a critical role of myotubularin in maintaining dendrite morphology and function during the cellular stress imposed by either oxidative stress or pathogen infection. Our study also provides insights into the role of dendrite degeneration in AWC neurons leading to the decrease in longevity upon pathogen exposure due to the hyperactivation of the DAF-16.

## Experimental procedures

### *C. elegans* strains and bacterial culture

The *C. elegans* strains used in this study are WT N2 Bristol, CX5974 [*kyIs262* [*unc-86::myr::gfp* + *odr-1::rfp*] IV], RB2048 [*mtm-10(ok2711)*], and they were procured from the *Caenorhabditis* Genetics Center (University of Minnesota). The following lines were generated in this study: AY161 [*kyIs262* [*unc-86::myr::gfp* + *odr-1::rfp*]; *mtm-10(ac270)*], AY162 [*kyIs262* [*unc-86::myr::gfp* + *odr-1::rfp*]; *mtm-10(ok2711)*], AY163 [acEx [P*mtm-10::mtm-10*+ *kyIs262* [*unc-86::myr::gfp* + *odr-1::rfp*]; *mtm-10 (ac270)*], AY164 [P*odr-1::mtm-10*+ *kyIs262* [*unc-86::myr::gfp* + *odr-1::rfp*]; *mtm-10(ac270)*], and AY165 [P*odr-1::rfp*+P*mtm-10::gfp*]. The strains were maintained at 20 °C and cultured on plates containing nematode growth medium (NGM).

The bacterial strains used were *E. coli* OP50 and *P. aeruginosa* PA14.

### Forward genetic screen for mutants susceptible to pathogen-mediated dendrite degeneration

In a previously published forward genetic screen for mutants susceptible to pathogen-mediated dendrite degeneration, 17 mutants were selected based on changes in the morphology of the dendrites that have been linked to degeneration (13). Briefly, mutagenized CX5974 animals expressing RFP in AWC and AWB neurons were allowed to develop to young adults on *E. coli*. These animals were observed under a fluorescent microscope, and mutants exhibiting any morphological changes in the dendrites were removed. The remaining mutants were transferred to plates containing *P. aeruginosa* and incubated for 24 h at 25 °C. The animals exhibiting any morphological changes in the dendrites after 24 h were transferred individually to separate plates. Around 80,000 haploid genomes were screened, and only the animals showing above 65% phenotype were backcrossed six times before further analysis (13).

### Fluorescence imaging

The fluorescent images were taken with a Zeiss Laser-Scanning Confocal microscope with Airyscan.2system (LSM-980) using grid-based optical and tile sectioning using a 20× and 40× objective water-immersion lens. The animals were immobilized with 20 mM sodium-azide in M9 buffer, mounted on a 2% agar pad, and covered with a glass coverslip. The glass coverslips were then coated with nail polish to seal them. The mutants were screened using a Leica M165 FC fluorescence microscope.

### Quantification of dendrite degeneration

Animals were visualized for morphological changes in the dendrites, which are considered hallmarks of neurodegeneration, including soma branching, wavy dendrites, dendrite branching, and beaded dendrites (23). Bacterial lawns were prepared by seeding the entire surface of modified NGM media 6-cm diameter plates with 300 μl of the 8 to 10 h culture of *P. aeruginosa* and incubated at 37 °C. The animals used were in the young adult stage. They were transferred to *P. aeruginosa* seeded plates and grown for 24 h at 25 °C as described (14). For microscopy, the animals were anesthetized with sodium-azide (20 mM) in M9 buffer, mounted onto agar pads, covered with a coverslip, followed by sealing with nail polish. The visualization was done using a Zeiss microscope or Leica M165 FC microscope. The criteria for the quantification of changes in the dendrite to be termed as degeneration was used as described previously (44, 45). The animals were only considered exhibiting dendrite degeneration if the morphological defects in bead-like structures were five or more in number. An animal was only considered for the bubble-like lesions and waviness defects if the defects were observed along the dendrite length. In some animals, two or more morphological defects were even observed simultaneously. This assay was carried out five times with 50 animals for both control and mutant animals. For calculating the percentage of animals exhibiting the dendrite degeneration phenotype, the number of animals showing dendrite degeneration was divided by the total number of animals in each group.

### Whole-genome sequencing and data analysis for the identification of *mtm-10*

For whole-genome sequencing, the DNA of AY161 mutant animals was extracted. Briefly, the mutants were grown at 20 °C on 10 cm NGM plates seeded with *E. coli* OP50. These animals were grown until starvation, and then the plates were rinsed thrice with M9 buffer to remove any bacteria. Then, the animals were incubated in M9 buffer with rotation for 2 h and washed three times with M9 buffer to remove bacteria from the intestine. The genomic DNA was extracted using the Gentra Puregene kit (Qiagen). The DNA was subjected to whole-genome sequencing on an Illumina HiSeq 4000 sequencing platform using 50 single-end nucleotide reads. DNA libraries were prepared according to a standard Illumina protocol. Library preparation and whole-genome sequencing were performed at Novogene Genomic Services & Solutions Company.

For analyzing the whole-genome sequence data, the EMS density mapping workflow from the Cloud Map program of the Galaxy web platform was used. A list of SNPs in the mutant was generated by comparing it with the reference *C. elegans* (WS220). After that, the common SNPs were subtracted, and the linkage map for the mutant was created (Fig. 1*B* and Table S1). To name *Y48G1C.10*, the online tool DRSC Integrative Ortholog Prediction Tool version 9 was used (25). This tool is used to predict orthologous genes among different animals, including humans, mice, zebrafish, *C. elegans*, *Drosophila*, and *Saccharomyces cerevisiae*. Based on the ortholog analysis score obtained (Fig. S4), gene *Y48G1C.10* was named *mtm-10*.

### Paraquat treatment

The animals were treated with paraquat according to a published protocol (46) with some modifications. Briefly, *E. coli* OP50 was grown in LB broth containing streptomycin (300 μg/ml) at 37 °C overnight, concentrated ten times, and plated onto NGM plates containing 5 mM paraquat (Sigma-Aldrich 856177) and 300 μg/ml streptomycin and incubated at room temperature overnight. Then, young adult animals were transferred to plates containing 5 mM paraquat and seeded with *E. coli* OP50. The animals were grown for 24 h at 20 °C. After 24 h, the animals were washed thrice with M9 buffer to eliminate any bacteria. The animals were then immobilized with 20 mM sodium-azide in M9 buffer, mounted on a 2% agar pad, and covered with a glass coverslip. The glass coverslips were then coated with nail polish to seal them. The visualization was carried out using a Zeiss microscope or Leica M165 FC microscope. This experiment was repeated thrice with 50 worms in each group and triplicate sets.

### *C. elegans* killing assay on *P. aeruginosa*

Animals for the assay were obtained by synchronization, allowing gravid animals to lay eggs for 4 to 6 h at 20 °C on NGM plates. The bacterial lawns were prepared by spreading 300 μl of 8 to 10 h grown culture of *P. aeruginosa* on the entire, complete surface of modified NGM agar medium (0.35% peptone) and incubated overnight at 37 °C. Plates were cooled to room temperature for at least 1 h before seeding them with synchronized young adult animals. The killing assays were performed at 25 °C, and live animals were transferred daily to fresh plates. The animals were scored every 12 h and were considered dead when they failed to respond to touch. Animals missing from the agar plate were censored on the day of loss. The Kaplan–Meier method was used to calculate the survival fractions, and a log-rank determined statistical significance between survival curves. Each experiment was performed thrice, and a total of 200 animals per condition were used unless otherwise indicated.

### Cloning and generation of transgenic *C. elegans* strains

For *mtm-10* rescue, a PCR fragment containing the 4 kb promoter and the *mtm-10* gene was generated and injected in strain AY161 along with the coinjection marker P*unc-122*::GFP. For the AWC neuron-specific expression of *mtm-10*, plasmid pRK1 (pPD95.77_SL2::GFP) was used to prevent gene products from fusing with GFP (47). The plasmid pSK1 (pPD95.77_P*odr-1*::*mtm-10*_SL2::GFP) was constructed by inserting 4 kb of the *odr-1* promoter sequence upstream into the *SphI* and *SalI* sites and *mtm-10* sequence into the *Xbal* and *Xmal* sites of the plasmid. Plasmid pSK1 was injected in strain AY161. For the identification of the foci of *mtm-10* expression, WT animals were injected with plasmid pSK2 constructed by cloning the 4 kb promoter of the *mtm-10* gene into the *SphI* and *SalI* sites of the pRK1 vector. Transgenic strains were created by injecting 20 to 30 ng/μl of the plasmids.

### Chemotaxis assay

The chemotaxis assay was performed to check the functionality of the AWC neurons as described previously (13) with some modifications. AWC are chemosensory neurons used in chemotaxis to volatile odorants, including isoamyl alcohol, benzaldehyde, and butanone. We used benzaldehyde as an odorant diluted with 95% ethanol (0.2% vol/vol) for this assay. Briefly, synchronized young adult animals were added to plates seeded with *P. aeruginosa* and incubated for 24 h at 25 °C. After the incubation, the animals were washed three times with M9 buffer to remove any bacteria, followed by washing with S basal twice. Then, the small volume of S basal was added to the washed animals, which were subsequently placed at the center of the chemotaxis plate. The chemotaxis plate was divided into four equal quadrants and a circle of 0.5 cm around the origin of the plate. Each quadrant was marked with either “T” for benzaldehyde or “C” for “95% Ethanol”, and these points were marked equidistant from the center. Once the washed animals were placed on the chemotaxis plate, they were allowed to move freely for 1 h, followed by adding 1 μl of sodium azide to the odor spots to anesthetize the animals at the respective spots. This assay was repeated six times in triplicates and on different days. For the chemotaxis index calculation, the number of animals in the “T” region minus the number of animals in the “C” region were divided by the total number of animals. The chemotactic index (CI) was calculated by the equation below:

$$\text{Chemotactic}\;\text{Index}\left(\text{CI}\right)=\frac{\left(\text{Total}\;\text{animals}\;\text{at}\;"\text{T"}\right)-\left(\text{Total}\;\text{animals}\;\text{at}\;\text{"C"}\right)}{\text{Total}\;\text{number}\;\text{of}\;\text{animals}}$$

### RNA isolation and quantitative real-time PCR

Animals were synchronized and placed on NGM plates seeded with *E. coli* OP50 and grown at 20 °C. The animals were grown until the young adult stage and then collected by washing three times with M9 buffer to remove any bacteria. These animals were added to modified NGM plates containing *P. aeruginosa* PA14 for 24 h at 25 °C. The *P. aeruginosa* plates were prepared by seeding the modified NGM plates with 300 μl of *P. aeruginosa* culture followed by overnight incubation at 37 °C. Animals were then washed off from these plates with M9 buffer 3 to 4 times and frozen in TRIzol (Life Technologies), followed by total RNA extraction using the RNeasy Plus Universal Kit (QIAGEN). For removing the residual genomic DNA, TURBO DNase (Life Technologies) was used, and 2 μg of total RNA was reverse transcribed with random primers using the High-Capacity cDNA Reverse Transcription Kit (Applied Biosystems). Quantitative RT-PCR was carried out using Power SYBR PCR Master Mix (Applied Biosystems) on an Applied Biosystems 7900HT real-time PCR machine in 96-well plate format using 25 μl in each reaction. The relative fold-changes of the transcripts were calculated using the comparative CT (2-ΔΔCT) method and normalized to pan-actin values obtained using the Step-OnePlus Software (Life Technologies). Three technical replicates were used, and the experiment was repeated three times.

### RNA interference

Loss-of-function phenotypes were generated by feeding the animals with *E. coli* strain HT115(DE3) expressing double-stranded RNA (dsRNA) homologous to a target gene. RNA interference (RNAi) was carried out as described previously (13). Briefly, *E. coli* carrying the appropriate vectors were grown overnight at 37 °C in LB broth containing ampicillin (100 mg/ml) and tetracycline (12.5 mg/ml). These bacteria were then plated onto NGM plates containing 100 mg/ml ampicillin and 3 mM IPTG (RNAi plates). RNA interference-expressing bacterial clones were grown for 12 to 14 h at 37 °C. Gravid adults were transferred to RNAi-expressing bacterial lawns and allowed to lay eggs for 2 to 4 h. The eggs were allowed to develop at 20 °C until young adults, and the young adults were then used for the pathogen exposure followed by lifespan assay. In all experiments, *unc-22* RNAi was included as a positive control to account for the RNAi efficiency, and the RNAi clones were sequenced before use. The RNAi clones were from the Ahringer RNAi library.

### *C. elegans* longevity assay

Lifespan assays were performed after the animals were infected with *P. aeruginosa* infection for 24 h at 25 °C. After infection, mutant or control animals grown on *P. aeruginosa* were rinsed by transferring them into 100 ml M9 containing 300 mg/ml of streptomycin on NGM plates seeded with *E. coli* OP50. The bacteria attached to the body wall was removed by allowing the animals to crawl out of the solution toward the *E. coli* lawn. Animals were further transferred onto new NGM plates supplemented with streptomycin (100 mg/ml), kanamycin (50 mg/ml), and nystatin (10 mg/ml) to avoid contamination. Plates were seeded with UV-killed *E. coli* as described earlier (29). The animals were scored as alive, dead, or gone each day, and live animals were transferred to fresh plates if needed. Animals that failed to display touch-provoked or pharyngeal movement were scored as dead (Table S2). Each experiment was repeated three times with 100 worms in each group. For the lifespan assay after RNAi, the animals were exposed to PA14 for 24 h at 25 °C and analyzed as described above. This experiment was repeated three times with 100 worms in each group. The assays were performed at 20 °C.

### Statistical analysis

Statistical analysis was performed using Graph Pad Prism 9 (Graph Pad Software). For survival, the Kaplan–Meier method was used to calculate the survival fractions, and statistical significance between survival curves was determined using the log-rank test. Survival curves were considered statistically significant when *p*-values were <0.05. Performed two-way ANOVA followed by Dunnett’s multiple comparisons post-hoc test for comparison between multiple groups and statistical significance, and *p*-values <0.05 were considered significant. Two-sample *t* test was also used when appropriate. Each experiment was repeated at least three times, and each comparison was made with the corresponding control group individually. Mean survival is shown in Table S2. In figures, all bars represent mean ± S.E.M. and *asterisks* (∗) denote *p*-value as ∗*p* < 0.05, ∗∗*p* < 0.01, ∗∗∗*p* < 0.001, and ∗∗∗∗*p* < 0.0001, ns, not significant.

## Data availability

All the data are contained within the article.

## Supporting information

This article contains supporting information.

## Conflict of interest

The authors declare that they do not have any conflicts of interest with the contents of this article.
