# Supplementary material for: Myotubularin-related protein protects against neuronal degeneration mediated by oxidative stress or infection
Source: J Biol Chem. 2022 Jan 29;298(3):101614. doi: 10.1016/j.jbc.2022.101614 (PMC8889260; doi:10.1016/j.jbc.2022.101614)
Supplement: Supplemental Table S1 [file mmc1.docx]

**Table S1. List of homozygous single nucleotide polymorphisms (SNPs) for the mutant after comparing it with the reference *C. elegans* (WS220)**

| **Chromosome** | **Position** | **Reference** | **Change** | **Quality** | **Gene_ID** | **Effect** | **Old AA/**  **New AA** | **Old codon/**  **New codon** | **Codon**  **Number**  **(CDS)** | **CDS size** |  | **CDS_size** | **Old_codon/New_codon** | **Codon_Num(CDS)** | **CDS_size** |
| --- | --- | --- | --- | --- | --- | --- | --- | --- | --- | --- | --- | --- | --- | --- | --- |
| I | 84500 | G | A | 48.77 | Y48G1C.10 | NS_CODING | G/R | Gga/Aga | 270 | 1221 |  |  | Gga/Aga | 270 | 1221 |
| I | 1466491 | G | A | 127.53 | F47G6.3 | NS_CODING | E/K | Gag/Aag | 67 | 1041 |  |  | Gag/Aag | 67 | 1041 |
| I | 4088480 | C | T | 48.77 | C09D1.1 | NS_CODING | S/F | tCt/tTt | 6993 | 22326 |  |  | tCt/tTt | 6993 | 22326 |
| I | 4088480 | C | T | 48.77 | C09D1.1 | NS_CODING | S/F | tCt/tTt | 948 | 4191 |  |  | tCt/tTt | 948 | 4191 |
| I | 4088480 | C | T | 48.77 | C09D1.1 | NS_CODING | S/F | tCt/tTt | 7633 | 24246 |  |  | tCt/tTt | 7633 | 24246 |
| I | 4088480 | C | T | 48.77 | C09D1.1 | NS_CODING | S/F | tCt/tTt | 945 | 4182 |  |  | tCt/tTt | 945 | 4182 |
| I | 4724458 | C | T | 207.17 | C10H11.5 | NS_CODING | T/I | aCa/aTa | 98 | 1581 |  |  | aCa/aTa | 98 | 1581 |
| I | 10267118 | C | G | 44.89 | ZC247.1 | NS_CODING | L/F | ttG/ttC | 3608 | 13170 |  |  | ttG/ttC | 3608 | 13170 |
| I | 10269037 | A | C | 275.13 | ZC247.1 | NS_CODING | F/V | Ttc/Gtc | 2969 | 13170 |  |  | Ttc/Gtc | 2969 | 13170 |
| II | 866039 | C | G | 38.39 | Y46B2A.3 | NS_CODING | R/T | aGa/aCa | 260 | 3438 |  |  | aGa/aCa | 260 | 3438 |
| II | 7367531 | T | C | 38.39 | F43E2.6 | NS_CODING | N/D | Aac/Gac | 63 | 291 |  |  | Aac/Gac | 63 | 291 |
| II | 7367531 | T | C | 38.39 | F43E2.6 | NS_CODING | N/D | Aac/Gac | 63 | 291 |  |  | Aac/Gac | 63 | 291 |
| II | 7367531 | T | C | 38.39 | F43E2.6 | NS_CODING | N/D | Aac/Gac | 71 | 315 |  |  | Aac/Gac | 71 | 315 |
| II | 7367536 | T | C | 38.39 | F43E2.6 | NS_CODING | D/G | gAt/gGt | 61 | 291 |  |  | gAt/gGt | 61 | 291 |
| II | 7367536 | T | C | 38.39 | F43E2.6 | NS_CODING | D/G | gAt/gGt | 61 | 291 |  |  | gAt/gGt | 61 | 291 |
| II | 7367536 | T | C | 38.39 | F43E2.6 | NS_CODING | D/G | gAt/gGt | 69 | 315 |  |  | gAt/gGt | 69 | 315 |
| II | 11215457 | A | G | 165.08 | T06D8.1 | NS_CODING | D/G | gAt/gGt | 2450 | 10833 |  |  | gAt/gGt | 2450 | 10833 |
| II | 11215457 | A | G | 165.08 | T06D8.1 | NS_CODING | D/G | gAt/gGt | 2450 | 11031 |  |  | gAt/gGt | 2450 | 11031 |
| II | 13546058 | C | T | 87.98 | Y48E1B.1 | NS_CODING | E/K | Gag/Aag | 225 | 1494 |  |  | Gag/Aag | 225 | 1494 |
| III | 440593 | C | T | 87.98 | Y55B1BR.1 | NS_CODING | S/L | tCa/tTa | 14 | 1527 |  |  | tCa/tTa | 14 | 1527 |
| III | 2502273 | G | A | 38.39 | Y54F10AM.7 | NS_CODING | A/T | Gcc/Acc | 39 | 555 |  |  | Gcc/Acc | 39 | 555 |
| IV | 7592875 | T | C | 48.77 | Y2C2A.1 | NS_CODING | S/P | Tct/Cct | 761 | 14703 |  |  | Tct/Cct | 761 | 14703 |
| IV | 7735099 | G | A | 117.15 | C50F7.2 | NS_CODING | G/D | gGt/gAt | 155 | 1677 |  |  | gGt/gAt | 155 | 1677 |
| IV | 10794037 | C | A | 48.77 | C06G8.2 | NS_CODING | F/L | ttC/ttA | 687 | 2358 |  |  | ttC/ttA | 687 | 2358 |
| IV | 15035402 | C | T | 48.77 | Y41E3.9 | NS_CODING | S/L | tCa/tTa | 704 | 2559 |  |  | tCa/tTa | 704 | 2559 |
| IV | 15035402 | C | T | 48.77 | Y41E3.9 | NS_CODING | S/L | tCa/tTa | 1292 | 4323 |  |  | tCa/tTa | 1292 | 4323 |
| IV | 16654872 | C | T | 87.98 | Y51H4A.12 | NS_CODING | G/E | gGa/gAa | 296 | 5034 |  |  | gGa/gAa | 296 | 5034 |
| IV | 17123467 | C | T | 87.98 | Y116A8C.36 | NS_CODING | P/L | cCt/cTt | 771 | 3258 |  |  | cCt/cTt | 771 | 3258 |
| IV | 17123467 | C | T | 87.98 | Y116A8C.36 | NS_CODING | P/L | cCt/cTt | 771 | 3258 |  |  | cCt/cTt | 771 | 3258 |
| V | 6174567 | G | T | 33.45 | W06H8.8 | NS_CODING | Q/K | Caa/Aaa | 7518 | 55689 |  |  | Caa/Aaa | 7518 | 55689 |
| V | 14105797 | G | C | 87.98 | D1086.9 | NS_CODING | E/Q | Gag/Cag | 515 | 2061 |  |  | Gag/Cag | 515 | 2061 |
| V | 20211155 | A | T | 33.45 | Y113G7B.12 | NS_CODING | D/E | gaT/gaA | 280 | 3129 |  |  | gaT/gaA | 280 | 3129 |
| X | 13550735 | A | G | 536.69 | R01E6.1 | NS_CODING | I/T | aTa/aCa | 7 | 3204 |  |  | aTa/aCa | 7 | 3204 |
| X | 13550736 | T | C | 736.13 | R01E6.1 | NS_CODING | I/V | Ata/Gta | 7 | 3204 |  |  | Ata/Gta | 7 | 3204 |
