# Supplementary material for: Myotubularin-related protein protects against neuronal degeneration mediated by oxidative stress or infection
Source: J Biol Chem. 2022 Jan 29;298(3):101614. doi: 10.1016/j.jbc.2022.101614 (PMC8889260; doi:10.1016/j.jbc.2022.101614)
Supplement: Supplemental Table S2 [file mmc2.docx]

**I. Mean survival of animals exposed to P. aeruginosa PA14**

| **Genotype^1^** | **Bacteria(strain)^2^** | **Mean survival ± SD (Hours)^3^** | **% Change in mean to Control** | **N(n) ^4^** | **Figure** |
| --- | --- | --- | --- | --- | --- |
| Control CX5974 (Parent strain) | *P. aeruginosa* (PA14) full lawn | 55.03±10.32 |  | 3(200) | Figure. 5A |
| *mtm-10(ac270)* | *P. aeruginosa* (PA14) full lawn | 52.65±10.40 | 0.08 | 3(200) |  |
| *mtm-10(ok2711)* | *P. aeruginosa* (PA14) full lawn | 52.19±9.22 | -1.09 | 3(200) |  |
| *mtm-10(ac270); Pmtm-10::mtm-10* | *P. aeruginosa* (PA14) full lawn | 53.80±9.87 | -0.44 | 3(200) |  |
| *mtm-10(ac270); Podr-1::mtm-10* | *P. aeruginosa* (PA14) full lawn | 53.45±6.53 | -3.78 | 3(200) |  |

**1**Genotype: Wild-type hermaphrodites or the indicated mutant/strain were analyzed.

**2**Bacteria: Bacterial species of the indicated strains were seeded on the NGM dish to form a lawn.

**3**Mean, % Change. Comparisons are to the matched control, which is the WT, control. The Kaplan-Meier method was used to calculate the survival fractions, and statistical significance between survival curves was determined using the log-rank test.

**4**N: Total number of hermaphrodites analyzed, and the number of independent experiments. Animals were synchronized by egg-laying and allowed to develop into young adults at 20ºC and all survival experiments were done with the indicated bacterial at 25ºC, except it is indicated.

**II. Mean lifespan of animals exposed to *P. aeruginosa* PA14 for 24hours and then on UV-killed *E. coli* OP50**

| **Genotype^1^** | **Bacteria(strain)^2^** | **Mean survival ± SD (Days)^3^** | **% Change in mean to Control** | **N(n) ^4^** | **Figure** |
| --- | --- | --- | --- | --- | --- |
| Control CX5974 (Parent strain) | UV-killed *E. coli* OP50 | 7.57±1.78 |  | 3(300) | Fig. 5B |
| *mtm-10(ac270)* | UV-killed *E. coli* OP50 | 4.63±0.16 | -1.62 | 3(300) |  |
| *mtm-10(ok2711)* | UV-killed *E. coli* OP50 | 4.73±0.23 | 0.076 | 3(300) |  |
| *mtm-10(ac270);Pmtm-10::mtm-10* | UV-killed *E. coli* OP50 | 7.61±1.24 | 1 | 3(300) |  |
| *mtm-10(ac270);Podr-1::mtm-10* | UV-killed *E. coli* OP50 | 7.99±1.88 | 0.64 | 3(300) |  |

**1**Genotype: Wild-type hermaphrodites or the indicated mutant/strain were analyzed.

**2**Bacteria: Bacterial species of the indicated strains were seeded on the NGM dish to form a lawn and then exposed to UV.

**3**Mean, % Change. Comparisons are to the matched control, which is the WT, control. The Kaplan-Meier method was used to calculate the survival fractions, and statistical significance between survival curves was determined using the log-rank test.

**4**N: Total number of hermaphrodites analyzed, and the number of independent experiments. Animals were synchronized by egg-laying and allowed to develop into young adults at 20ºC and all survival experiments were done with the indicated bacterial at 20ºC, except it is indicated.

**III. Mean lifespan of animals exposed to *daf-16 RNAi and Control RNAi then on UV-killed E. coli OP50***

| **Genotype^1^** | **Bacteria(strain)^2^** | **Mean survival ± SD (Days)^3^** | **% Change in mean to Control** | **N(n) ^4^** | **Figure** |
| --- | --- | --- | --- | --- | --- |
| CX5974 (Parent strain)-Control RNAi | UV-killed E. coli OP50 | 7.76±0.42 |  | 3(300) | Fig. 5D |
| CX5974 (Parent strain)-daf-16 RNAi | UV-killed E. coli OP50 | 4.11±0.46 | 0.040375534 | 3(300) |  |
| mtm-10(ac270)-Control RNAi | UV-killed E. coli OP50 | 3.65±0.36 | -0.054648043 | 3(300) |  |
| mtm-10(ac270)-daf-16 RNAi | UV-killed E. coli OP50 | 6.64±0.81 | 0.391957545 | 3(300) |  |
