# Supplementary material for: Myotubularin-related protein protects against neuronal degeneration mediated by oxidative stress or infection
Source: J Biol Chem. 2022 Jan 29;298(3):101614. doi: 10.1016/j.jbc.2022.101614 (PMC8889260; doi:10.1016/j.jbc.2022.101614)
Supplement: Supplemental Figures S1–S5 [file mmc3.pdf]

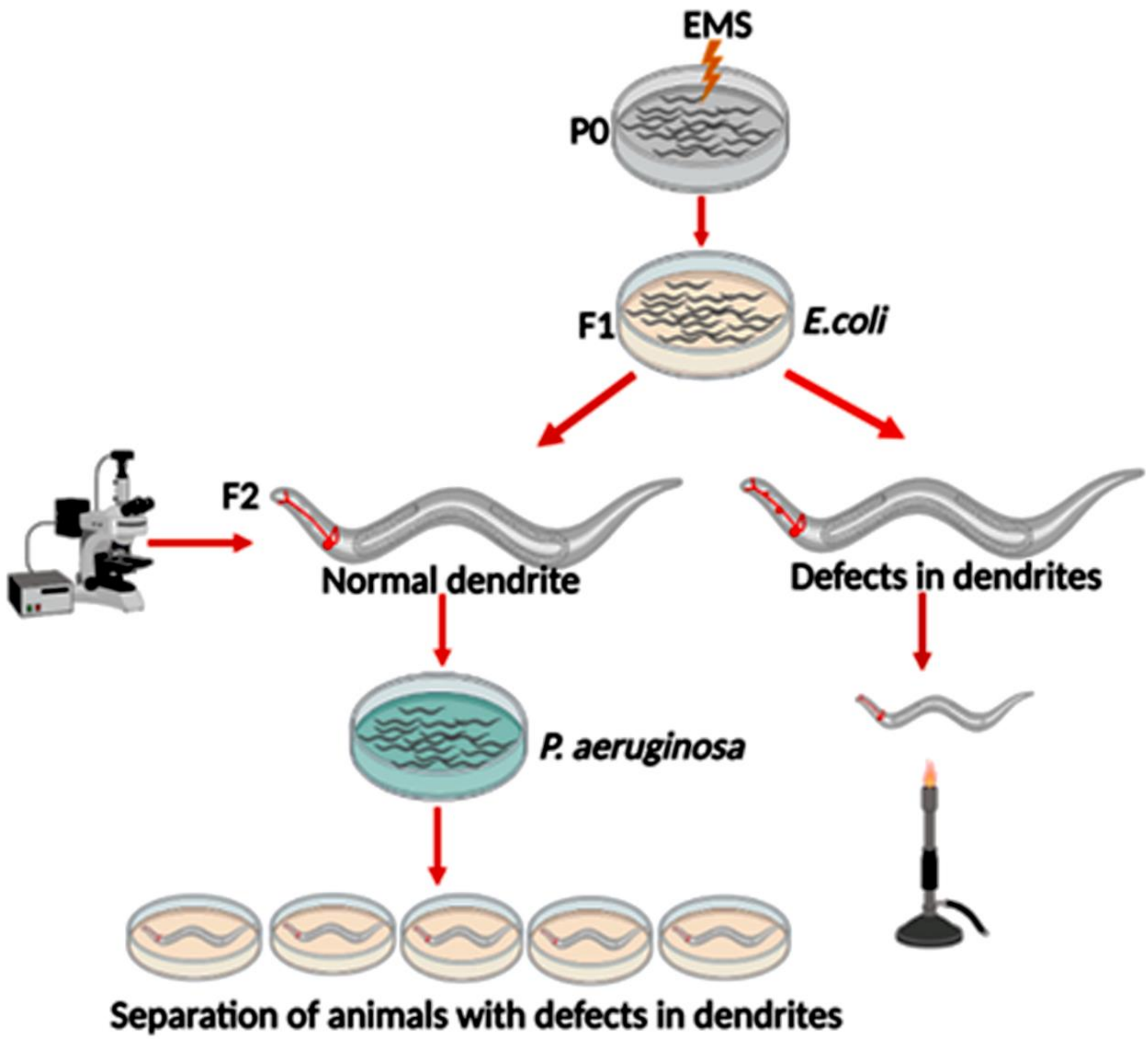

Figure S1. Schematic representation of the forward genetic screen. Animals (P0) were treated with ethylmethanesulfonate (EMS) and the animals of the F1progeny were self-fertilized. Then, dendrites of F2 animals were analyzed for degeneration, and animals showing any changes were discarded. The mutant animals with normal dendrites were infected with *P. aeruginosa* for 24 h and then screened for dendrite degeneration.

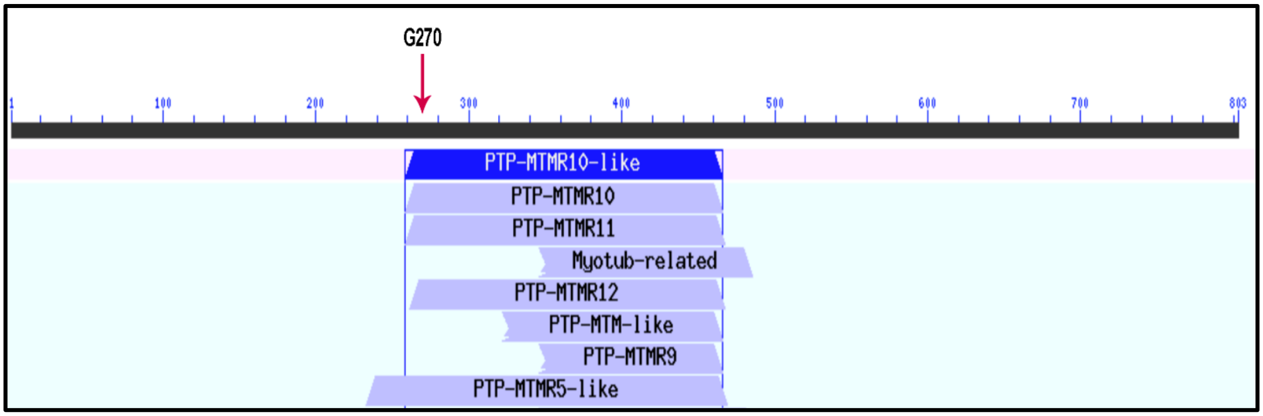

Figure S2. The mutation in the *mtm-10* gene G270A from glycine(G) to arginine(A) at residue 270 is in the conserved PTP-MTMR10-like domain.

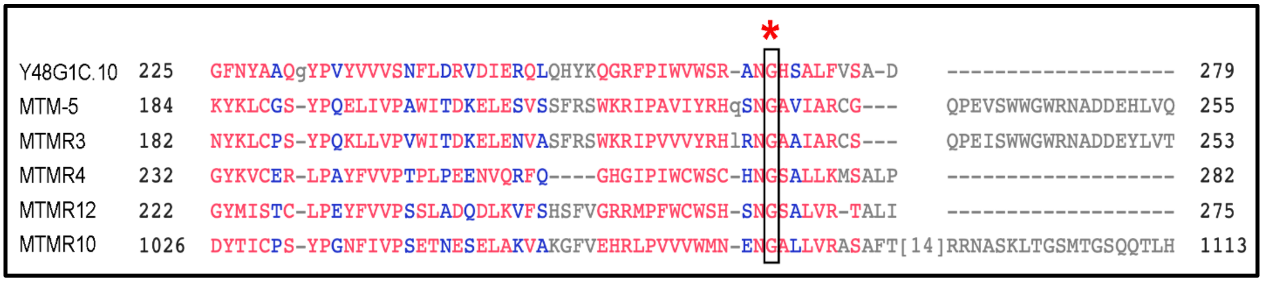

Figure S3. The mutation site G270 is conserved among several MTMR family proteins.

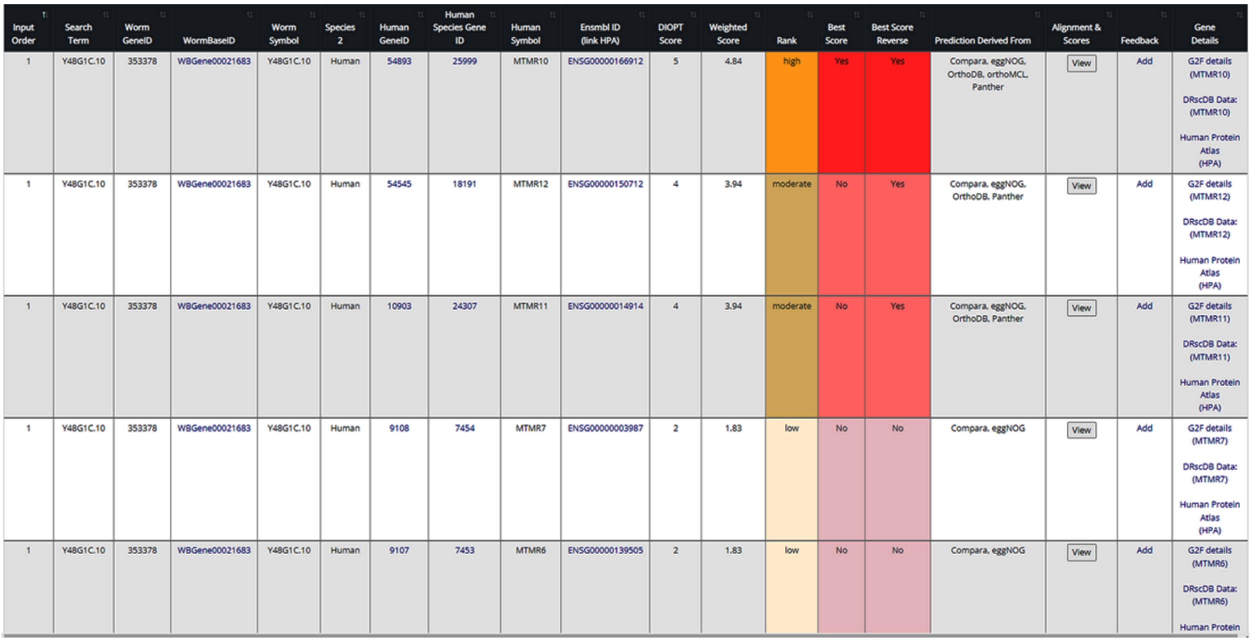

Input Order	Search Term	Worm GeneID	WormBaseID	Worm Symbol	Species 2	Human GeneID	Human Species Gene ID	Human Symbol	Ensmbl ID (link HPA)	DIOPT Score	Weighted Score	Rank	Best Score	Best Score Reverse	Prediction Derived From	Alignment & Scores	Feedback	Gene Details
1	Y48G1C.10	353378	WBGene00021683	Y48G1C.10	Human	54893	25999	MTMR10	ENSG00000166912	5	4.84	high	Yes	Yes	Compara, eggNOG, OrthoDB, orthoMCL, Panther	View	Add	G2F details (MTMR10) DRscDB Data: (MTMR10) Human Protein Atlas (HPA)
1	Y48G1C.10	353378	WBGene00021683	Y48G1C.10	Human	54545	18191	MTMR12	ENSG00000150712	4	3.94	moderate	No	Yes	Compara, eggNOG, OrthoDB, Panther	View	Add	G2F details (MTMR12) DRscDB Data: (MTMR12) Human Protein Atlas (HPA)
1	Y48G1C.10	353378	WBGene00021683	Y48G1C.10	Human	10903	24307	MTMR11	ENSG00000014914	4	3.94	moderate	No	Yes	Compara, eggNOG, OrthoDB, Panther	View	Add	G2F details (MTMR11) DRscDB Data: (MTMR11) Human Protein Atlas (HPA)
1	Y48G1C.10	353378	WBGene00021683	Y48G1C.10	Human	9108	7454	MTMR7	ENSG00000003987	2	1.83	low	No	No	Compara, eggNOG	View	Add	G2F details (MTMR7) DRscDB Data: (MTMR7) Human Protein Atlas (HPA)
1	Y48G1C.10	353378	WBGene00021683	Y48G1C.10	Human	9107	7453	MTMR6	ENSG00000139505	2	1.83	low	No	No	Compara, eggNOG	View	Add	G2F details (MTMR6) DRscDB Data: (MTMR6) Human Protein

Figure S4. Orthology analysis of the gene *Y48G1C.10* with the human *MTMR* (myotubularin related proteins genes using the online tool DRSC Integrative Ortholog Prediction Tool (DIOPT).

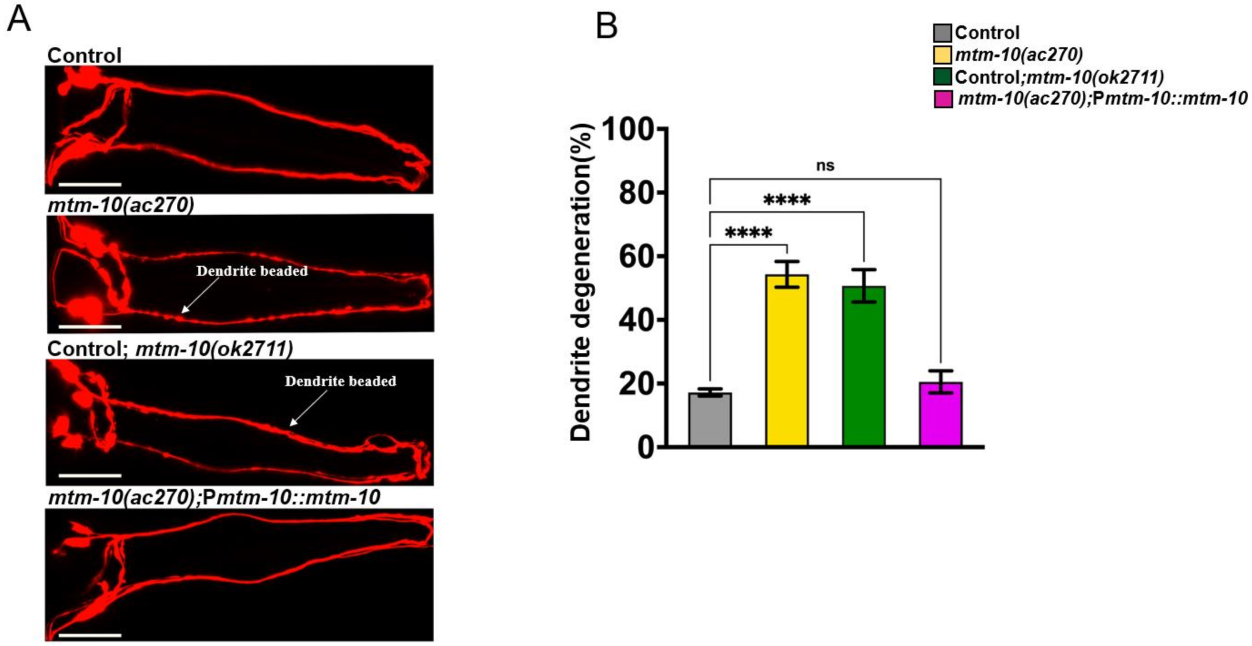

Figure S5. (A) Representative photomicrographs of dendrite degeneration in control CX5974 (Parent strain), *mtm-10 (ac270*), *mtm-10(ok2711),* and *mtm-10(ac270)*;P*mtm-10::mtm-10* animals expressing RFP in AWC after exposure to paraquat(5mM) for 24 hours. The scale bars represent 10 µm. Different morphological changes in the dendrites are indicted with white arrows. **(B)** Quantification of the dendrite degeneration phenotype in control, *mtm-10(ac270*), *mtm-10(ok2711),* and *mtm-10(ac270)*; P*mtm-10::mtm-10* animals expressing RFP in AWC neurons. N=3, number of animals=150. Control vs. *mtm-10(ac270*) (p<0.0001), *mtm-10 (ac270*) vs. *mtm-10(ac270*);P*mtm-10::mtm-10* (p<0.0001) via one-way ANOVA test.
