# Supplementary material for: Myotubularin-related protein protects against neuronal degeneration mediated by oxidative stress or infection
Source: J Biol Chem. 2022 Jan 29;298(3):101614. doi: 10.1016/j.jbc.2022.101614 (PMC8889260; doi:10.1016/j.jbc.2022.101614)
Supplement: Supplemental Data [file mmc4.docx]

| Gene Name | Forward primer sequence (5’-3’)  **Primers for Real Time qPCR** | Reverse primer sequence (5’-3’) |
| --- | --- | --- |
| Pan-*act* | TCGGTATGGGACAGAAGGAC | CATCCCAGTTGGTGACGATA |
| *dod-20* | CAAAGCTCATGCACCGTACAA | CGAGGAGTGTTACGTCTGCT |
| *dod-22* | CACCGAAACTATCAACGGCG | GCCATAGTTGCTTCGTCATCC |
| *spp-1* | CTCTCGTCGAGGGTGGAGA | ATGCAACGGCAACAGCATAG |
| *mtm-10* | ACCCATTTGGTGAACGGCTACTC | GTCGGCATGACGTCAGTTCTCAT |
| *gpd-2* | CAACCCACACTTCGTCAAGCTC | CTAGGTGAAAGTAGGATGAGACAGC |
| *dod-17* | AGTTGTTTGGTGGCCACTTCCA | GTGATGCCACCACCTTCACTGT |
| *dod-24* | TGGCATCCTTTGCTCTTGCAGT | TGAGCTCTGTCCATCCCATCCA |
| *k10D11.5* | CTGTCAGGCTGGCCACTTCAAT | TGGAAACAATGGTGGCTCGGTT |
| *sodh-1* | GAAGGAGCTGGAAGTGTTGTTC | CTCCACGTATAGTGAGGTACTCCTG |
